# Diagnostic accuracy of novel mRNA blood biomarkers of infection to predict outcomes in emergency department patients with undifferentiated abdominal pain

**DOI:** 10.1038/s41598-023-29385-3

**Published:** 2023-02-09

**Authors:** Andrew C. Meltzer, Richard S. Wargowsky, Seamus Moran, Tristan Jordan, Ian Toma, Tisha Jepson, Shiyu Shu, Yan Ma, Timothy A. McCaffrey

**Affiliations:** 1 Department of Emergency Medicine, School of Medicine and Health Sciences, The George Washington University Medical Center, Washington, DC 20037 USA; 2 Division of Genomic Medicine, Department of Medicine, The George Washington University Medical Center, Washington, DC 20037 USA; 3 True Bearing Diagnostics, Washington, DC 20037 USA; 4 Department of Biostatistics, The George Washington University Milken School of Public Health, Washington, DC 20037 USA; 5 Department of Biostatistics, University of Pittsburgh, Pittsburgh, PA 15260 USA

**Keywords:** Biomarkers, Diseases, Gene expression analysis

## Abstract

Abdominal pain represents greater than 20% of US Emergency Department (ED) visits due to a wide range of illnesses. There are currently no reliable blood biomarkers to predict serious outcomes in patients with abdominal pain. Our previous studies have identified three mRNA transcripts related to innate immune activation: alkaline phosphatase (ALPL), interleukin-8 receptor-β (IL8RB), and defensin-1 (DEFA1) as promising candidates to detect an intra-abdominal infection. The objective of this study was to evaluate the accuracy of these mRNA biomarkers to predict likely infection, hospitalization and surgery in Emergency Department patients with undifferentiated abdominal pain. We prospectively enrolled Emergency Department patients with undifferentiated abdominal pain who received an abdominal CT scan as part of their evaluation. Clinical outcomes were abstracted from the CT scan and medical records. mRNA biomarker levels were calculated independent of the clinical outcomes and their accuracy was assessed to predict infectious diagnoses, surgery and hospital admission. 89 patients were enrolled; 21 underwent surgery; 47 underwent hospital admission; and, no deaths were observed within 30 days. In identifying which cases were likely infectious, mRNA biomarkers’ AUC values were: ALPL, 0.83; DEFA1 0.51; IL8RB, 0.74; and ALPL + IL8RB, 0.79. In predicting which Emergency Department patients would receive surgery, the AUC values were: ALPL, 0.75; DEFA1, 0.58; IL8RB, 0.75; and ALPL + IL8RB, 0.76. In predicting hospital admission, the AUC values were: ALPL, 0.78; DEFA1, 0.52; IL8RB, 0.74; and, ALPL + IL8RB, 0.77. For predicting surgery, ALPL + IL8RB’s positive likelihood ratio (LR) was 3.97; negative LR (NLR) was 0.70. For predicting hospital admission, the same marker’s positive LR was 2.80 with an NLR of 0.45. Where the primary cause for admission was a potentially infectious disorder, 33 of 34 cases (97%) had positive RNA scores. In a pragmatic, prospective diagnostic accuracy trial in Emergency Department patients with undifferentiated abdominal pain, mRNA biomarkers showed good accuracy to identify patients with potential infection, as well as those needing surgery or hospital admission.

## Introduction

Abdominal pain represents more than 20% of annual US Emergency Department (ED) visits and the severity of the underlying illness ranges from minor to extreme^1^. Because identification of higher severity illness can be challenging, the Emergency Department work-up for abdominal pain is typically intense with blood tests in 70% and CT imaging in 30% of cases^1^. Major outcomes for Emergency Department patients with abdominal pain include hospital admission, abdominal surgery, Emergency Department return visits, and death^3^. Unlike the troponin biomarker for chest pain, there are no reliable serum biomarkers to predict major outcomes for abdominal pain^4^. An accurate biomarker for intra-abdominal infection and associated major outcomes would significantly improve diagnostic acumen. Past research with biomarkers procalcitonin, lactate and c-reactive protein and molecular biomarkers of abdominal sepsis have shown promise and limitations^5,6^. The mRNA biomarkers alkaline phosphatase (ALPL), interleukin-8 receptor-β (IL8RB), and neutrophil defensin α1 (DEFA1) mRNAs merit an examination of their diagnostic accuracy to identify intra-abdominal infections after prior research showed them as being markedly altered in patients with CT-confirmed appendicitis^7^. The ALPL and IL8RB markers were principally elevated in cases of abdominal infections such as appendicitis, that are thought to be biofilm infections. Conversely, the DEFA1 biomarker was highly elevated in typical bacterial infections and COVID-19 infection^8^. The objective of this study was to assess the accuracy of these mRNA biomarkers to identify likely infections and predict the need for hospitalization and surgery in Emergency Department patients with undifferentiated abdominal pain.

## Materials and methods

### Screening and enrollment

In this prospective cross-sectional study, novel mRNA transcripts were assessed in Emergency Department patients with undifferentiated abdominal pain to predict a likely infectious process, hospital admission or surgery. The study was designed to enroll consecutive patients presenting with abdominal pain at The George Washington University Hospital ED, a level-one trauma center and academic urban Emergency Department that treats approximately 80,000 patients per year. Study participants were screened from the larger pool of Emergency Department patients by trained professional research assistants for a chief complaint of abdominal pain or a related symptom. To qualify, participants must have met the following inclusion criteria: age greater than or equal to 18, presence of abdominal pain or discomfort, recipient of an abdominal CT scan performed during the index Emergency Department visit. Major exclusion criteria included abdominal symptoms lasting greater than or equal to 14 days, hemodynamic instability, previous diagnosis of an intra-abdominal infection within the past 30 days, inability to provide informed consent, prisoner or ward of state, recipient of intravenous contrast for a CT scan within prior 24 h, current regular recipient of hemodialysis, non-English speaking, Crohn’s disease, AIDS, lupus, rheumatoid arthritis, solid organ transplant, active chemotherapy, regular user of oral steroid medication or other systemic immunosuppressive agent. Enrollment occurred from September 2020 to September 2021, Monday through Friday, between the hours of 10 am and 10 pm by Emergency Department research staff. Sample size was set at 100 and was based on convenience and budgeted staffing not statistical calculations. Staffing was interrupted on multiple occasions due to COVID-19 safety protocols and staff absence. Informed consent was obtained for all patients and the protocol was approved by The George Washington University Institutional Review Board (IRB). All study protocol was performed in accordance with STARD guidelines and regulations, including conducting the biomarker assays without knowledge of the clinical diagnosis (see checklist, supplementary A). All methods were carried out in accordance with relevant guidelines and regulations and reviewed by the aforementioned IRB.

### RNA isolation

After the signed informed consent was obtained, venipuncture was performed in the Emergency Department to draw whole blood into an RNA preservative solution (Tempus). The blood tubes were allowed to incubate at room temperature (23 °C) for 2 h after collection, then immediately frozen at − 80 °C for later RNA isolation in batches of 20. The RNA isolation was subsequently performed via the Tempus Spin RNA Isolation Kit with on-column Turbo DNase (0.01 U/μL) (Thermo Fisher). The RNA yield was assessed by optical density at 280 nm using a NanoDrop-1000 spectrophotometer. The RNA's quality was assessed on an Agilent 2100 BioAnalyzer. mRNA was stored at − 80 °C. Personnel performing the assays were blinded to the subjects’ clinical characteristics and outcomes.

### Droplet digital PCR

DNAse-treated RNA was analyzed for mRNA levels of the biomarkers by ddPCR (Bio Rad), using gene-specific primers for the reverse transcriptase step (iScript). The measured copy number of each RNA biomarker was normalized to a percent of the ACTB copies per sample. The three mRNA biomarkers tested were DEFA1, ALPL and IL8RB plus a composite test: ALPL + IL8RB. Cut-offs for positivity were established prior to the beginning of the study for biomarker tests: DEFA > 10% and ALPL + IL8RB > 20% of the ACTB copies per sample. An overall mRNA positive status was computed as either DEFA1 > 10% or ALPL + IL8RB > 20%.

### Clinical measures

Final diagnoses and clinical outcomes were determined by reviewing each patient’s Emergency Department abdominal CT scan and medical record data. Data was first abstracted on the day of enrollment from the ED’s EHR (Electronic Health Record) system (Cerner). The EHR was searched again 30 days later for any Emergency Department return visits, for additional information about hospital course, and for surgical reports and/or death. Chart abstraction was performed by research assistants blinded to the results of the biomarker test. All records were reviewed by a minimum of two trained abstractors using structured data sheets for final diagnosis, for abdominal surgery, for hospital admission, for Emergency Department return visits, for death, and for other markers of care such as the need for antibiotics or intravenous fluids. Any discrepancies were resolved by a third investigator. In general, interventional radiology procedures (except simple biopsy) were considered with traditional general surgery procedures such as laparotomy as evidence of surgery. An expert panel of emergency medicine physicians further categorized their diagnoses by the likelihood of an infectious cause based on clinical knowledge and experience. In addition, mRNA biomarkers were compared to traditional tests of infection including white blood cell (WBC) count, neutrophil-to-lymphocyte (NLR) ratio and lactate using the Pearson correlation coefficient. The percent of patients positive for infection by mRNA (either DEFA1 > 10% or ALPL/IL8RB > 20%) was compared to the percent of patients positive by WBC count (defined as greater than 12 × 10^4^/mcl), the percent positive by neutrophil–lymphocyte ratio (NLR) (defined as > 6), and the percent of patients positive by lactate (defined as > 2 mM).

### Analysis plan

In descriptive analysis, median and interquartile range were calculated for continuous variables and frequency and percentage were calculated for discrete variables. Sensitivity, specificity, positive predictive value, negative predictive value, positive likelihood ratio and negative likelihood ratio were used to assess the accuracy of the categorized/dichotomized biomarkers in predicting surgery and hospital admission. Finally, we calculated the area under the AUC values for all three biomarkers when considered as continuous variables. All statistical analyses were conducted using R packages dplyr, tidyverse, pROC, caret and gtsummary. Full protocol available on request from corresponding author.

### Conference presentation

Abstract, Society of Academic Emergency Medicine, 2022, New Orleans, LA.

### Ethical approval and consent to participate

All subjects gave written informed consent under IRB Protocol #NCR191353, approved by The George Washington University Institutional Review Board.

## Results

**Figure 1:**
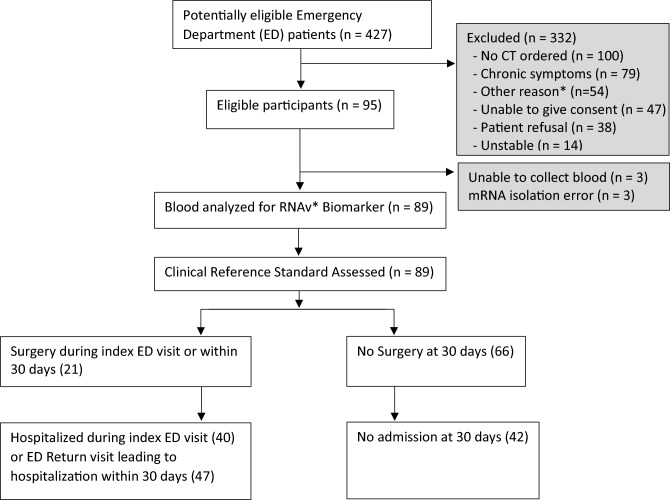
Overall study design. *RNAv Biomarker refers to the target biomarkers for the study: DEFA1, ALPL and IL8RB. *ED* Emergency Department.

A total of 427 Emergency Department patients were screened for participation in the study, and 95 patients that met the inclusion and exclusion criteria were consented. Six patients were not included in final analysis due to either an insufficient mRNA yield or inability to draw blood (Fig. 1). For the 89 patients enrolled, we calculated baseline characteristics and measured standard biomarkers such as WBC with differential counts and lactic acid levels (lactate) performed as part of normal clinical practice (Table 1). In total, the wide range of diagnoses reflected the heterogenous nature of undifferentiated abdominal pain in the Emergency Department including acute appendicitis (n = 10), cystitis and pyelonephritis (n = 8), bowel obstruction (n = 7), acute diverticulitis (n = 6), colitis (n = 6), obstructive renal stones (n = 6), and appendagitis (n = 2) (Table 2). Several patients with abdominal CT scans that did not show a cause for pain were categorized as “no organic cause of pain” (n = 24); these diagnoses included non-obstructive renal stones, constipation, gastritis, nausea, and vomiting. Of the 89 eligible patients, 21 underwent surgery (20 on index visit and one on return visit within 30 days), 47 underwent hospital admission (40 on index visit, seven who returned to the Emergency Department within 30 days after discharge), and no deaths were observed within 30 days.

**Table 1:** Summary of participants.

	All patients (N = 89)
Demographics	
Age (median, IQR (interquartile range))	45 (20, 86)
Black (n, %)	47, 53%
Hispanic (n, %)	4, 4.5%
Female (n, %)	68, 76%
Labs and vital signs	
Triage Temp (median, IQR)	97.3 (96.8, 97.9)
Triage Temp (> 100.3) (n, %)	7, 7.9%
WBC (white blood cell) (10^3^/mcl), (median, IQR)	9.6 (7.1, 11.8)
WBC > 12 × 10^3^/mcl (n, %)	20, 22.5%
Absolute lymphocyte count (10^3^/mcl), (median, IQR)	1.66 (1.27, 2.12)
Percent (%) lymphocytes, (median, IQR)	19 (12, 27)
Absolute neutrophil count (10^3^/mcl), (median, IQR)	6.8 (4.0, 9.5)
Percent (%) neutrophils, (median, IQR)	70 (62, 80)
NLR (neutrophil lymphocyte ratio) > 1.65 (n, %)	74 (83.1%)
Bands > 0 (n, %)	5 (5.6%)
Hemoglobin value (g/dl), (median, IQR)	13.1 (8.2, 16.9)
Platelet count (10^3^/mcl), (median, IQR)	274 (229, 332)
Lactic acid level (mmol/L), (median, IQR)	0.00 (0.00, 1.00)
> 2.0 (n, %)	4 (4.5%)
> 4.0 (n, %)	1 (1.1%)
Inflammatory bowel disease (n, %)	1 (1.1%)
HIV (human immunodeficiency virus) positive (n, %)	4 (4.5%)
RNA	
RNA yield (mcg/mL blood) (median, IQR)	11 (8, 15)
RNA integrity, scaled 0 to 10 (most integrity), (median, IQR)	8.7 (8.4, 9.2)
ACTB copies, (median, IQR)	170,000 (132,200, 208,000)
Outcomes (n, %)	
Hospital admission at index visit	40 (45%)
Any Emergency Department return visit within 30 days	19 (21%)
Emergency Department return visit leading to admission ≤ 30 days of Index	7 (7.9%)
Surgery during Index visit or ≤ 30 days of Index visit	21 (24%)
Died ≤ 30 days of Index visit	0 (0%)

**Table 2:** Summary of abdominal pain cases by infection likelihood and percent positive for ALPL/IL8R > 20% and DEFA1 > 10% biomarker.

Diagnosis	Total cases	ALPL/IL8RB > 20% (n, %)	DEFA1 > 10% (n, %)	ALPL/IL8R or DEFA1 positive (n, %)
Likely infectious	30	24 (80%)	8 (27%)	28 (93%)
Appendicitis	10	9 (90%)	1 (10%)	10 (100%)
Cystitis/pyelonephritis	8	6 (75%)	4 (50%)	8 (100%)
Diverticulitis	6	4 (67%)	1 (17%)	5 (83%)
Acute cholecystitis	1	1 (100%)	1 (100%)	1 (100%)
Intrabdominal abscess	1	1 (100%)	0	1 (100%)
Peritonitis (from any cause)	1	1 (100%)	0	1 (100%)
Pneumonia	1	1 (100%)	1 (100%)	1 (100%)
Genitalia tract infection	1	1 (100%)	0	1 (100%)
Cellulitis	1	0	0	0
Uncertain infectious	23	13 (57%)	6 (26%)	15 (65%)
Bowel obstruction (complete)	7	4 (57%)	3 (43%)	4 (57%)
Obstructive urinary stone	6	5 (83%)	0	5 (83%)
Enteritis/colitis/ileitis/duodenitis	6	1 (17%)	1 (17%)	2 (33%)
Epiploic appendagitis	2	1 (50%)	1 (50%)	2 (100%)
Pancreatitis	2	2 (100%)	1 (50%)	2 (100%)
Unlikely infectious	36	10 (28%)	7 (19%)	14 (39%)
No organic etiology for pain on CT	24	6 (25%)	5 (21%)	10 (42%)
Ruptured ovarian cyst	6	2 (33%)	1 (17%)	2 (33%)
Uterine fibroids	3	1 (33%)	0	1 (33%)
Abdominal mass NOS	1	1 (100%)	1 (100%)	1 (100%)
Cholelithiasis	1	0	0	0
Ovarian torsion	1	0	0	0
Total	89	47 (53%)	21 (24%)	57 (64%)

**Figure 2:**
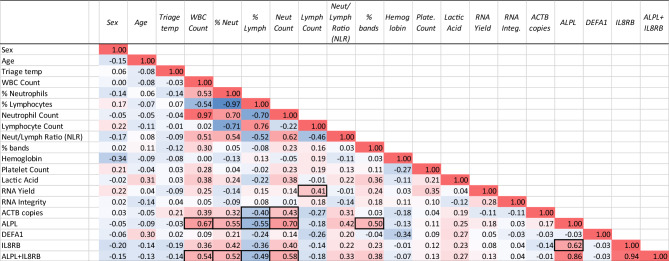
Correlation of clinical factors and biomarkers. Across all patients, regardless of clinical diagnosis, the relationship between the measured variables was explored by a correlational analysis to examine covariate trends. Correlations were calculated by the Pearson correlation coefficient (r), which is a measure of any linear association between two variables. Negative trends are highlighted in blue, and positive trends are highlighted in red. The value of r ranges between − 1 and 1.

In correlational analysis across all patients, regardless of diagnosis, the ALPL + IL8RB biomarker demonstrated strong associations with percent ALPL and WBC count (0.67), neutrophil count (0.70), and percent bands (0.50) (Fig. 2). The DEFA1 biomarker did not show notable correlations to any other metrics. There was a modestly positive relationship between lymphocyte count and total RNA yield (0.41), and ACTB copies to the neutrophil count (0.43) (Fig. 2).

### Risk of infectious etiology

**Figure 3:**
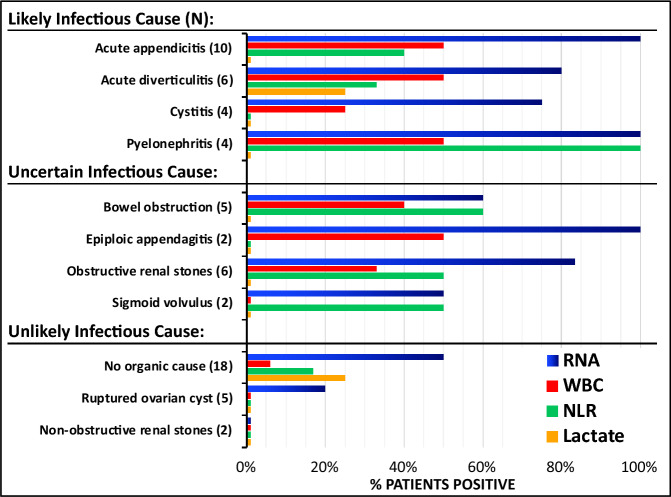
Comparison of RNA biomarkers with traditional biomarkers (WBC, NLR, Lactate). The clinical, imaging, and laboratory values of patients presenting with abdominal pain at the Emergency Department was used to categorize the patients into the diagnostic groups shown on the Y-axis. The percent of those patients that were positive for the novel RNA biomarkers DEFA1 > 5% or ALPL + IL8RB > 20% (BLUE bars), WBC count > 10k/μL (RED bars), neutrophil/lymphocyte ratio > 6 (NLR, GREEN bars), and lactate levels > 2 mM in plasma (YELLOW bars) are shown on the horizontal axis. Lactate was not ordered on all patients, but only one diagnosis (obstructive renal stones) had no cases where lactate was ordered.

RNA scores were more frequently positive in patients categorized with a likely infectious etiology (28/30, p = 7.75 × 10^−6^) or uncertain infectious etiology (15/23, p = 0.044) versus unlikely infectious etiology (14/36). RNA positive tests, defined as IL8RB + ALPL > 20% or DEFA1 > 10%, were more common in patients with a likely infectious etiology than WBC, NLR and lactate (Fig. 3). In likely infectious causes, the AUC values of the mRNA transcripts ALPL, DEFA1, IL8RB and ALPL + IL8R were 0.83, 0.51, 0.74 and 0.79 (Table 3).

**Table 3:** AUC values to assess accuracy of biomarkers as continuous variables to predict outcomes in emergency department abdominal pain.

	ALPL	DEFA1	IL8RB	ALPL/IL8RB
Likely infection	0.83	0.51	0.74	0.79
Abdominal surgery	0.75	0.58	0.75	0.76
Hospital admission	0.78	0.52	0.74	0.77

### Antibiotic use

A secondary analysis was conducted to determine if mRNA scores predicted the use of antibiotics prescribed as part of normal clinical practice. Overall, of the 89 included patients, 47 (52%) received antibiotics related to their abdominal pain. Of these 47, 39 patients (82.9%) had positive RNA scores. Of the 42 patients that did not receive antibiotics, 17 (40.5%) had positive RNA scores. Of the eight cases ‘missed’ by the RNA tests, two were categorized as “likely infectious”, five were “uncertain infectious”, and one was “unlikely infectious”. In a subset of the likely infection group who received antibiotics (n = 29), 27 (93%) had positive RNA scores. Conversely, in the subset of the unlikely infection group who did not receive antibiotics (n = 29), 21 (70%) had negative RNA scores (*p* = 0.041).

### Risk of hospital admission and surgery

An analysis was conducted to determine whether the mRNA levels would predict whether a patient required surgery relevant to the Emergency Department visit. Of the 21 surgical patients, 19 had positive RNA scores (90.5%). Using the RNA to predict the need for surgery, the AUC values were calculated for ALPL as 0.75; DEFA1, 0.58; IL8RB, 0.75; and ALPL + IL8RB, 0.76 (Table 3). When analyzed using a pre-established threshold, ALPL + IL8RB > 20%, the biomarker identified infection, need for surgery and hospital admission with positive likelihood ratios of 4.25, 3.97, 2.80 and negative likelihood ratios of 0.30, 0.70, 0.45, respectively (Table 4). When ALPL + IL8RB > 20% was combined with a DEFA1 > 10%, the positive predictive value became more pronounced for the outcomes of likely infection (0.90), surgery (0.86) and hospital admission (0.83) (Table 4).

**Table 4:** Comparison of biomarker accuracy as categorical variables per outcome in emergency department patients with abdominal pain.

RNA biomarker	Sensitivity	Specificity	Positive predictive value (PPV)	Negative predictive value (NPV)	Positive likelihood ratio (LR)	Negative likelihood ratio (LR)
A. Likely infection (n = 30) versus unlikely infection (n = 36)						
DEFA1 > 10%	0.53	0.57	0.27	0.81	1.24	0.82
ALPL/IL8RB > 20%	0.75	0.82	0.80	0.78	4.25	0.30
DEFA1 > 10% OR (ALPL/IL8RB) > 20%	0.68	0.89	0.90	0.64	5.85	0.37

RNA biomarker	Sensitivity	Specificity	PPV	NPV	Positive LR	Negative LR
B. Abdominal surgery (n = 21)						
DEFA1 > 10%	0.19	0.75	0.19	0.75	0.76	1.08
ALPL/IL8RB > 20%	0.37	0.91	0.81	0.57	3.97	0.70
DEFA1 > 10% OR (ALPL/IL8RB) > 20%	0.32	0.91	0.86	0.44	3.54	0.75
C. Hospital admission (n = 47)						
DEFA1	0.43	0.54	0.23	0.76	0.94	1.05
ALPL/IL8RBc	0.65	0.77	0.75	0.67	2.80	0.45
DEFA1 > 10% OR (ALPL/IL8RB) > 20%	0.59	0.79	0.83	0.53	2.78	0.52

Of the 40 (44.9%) patients admitted to the hospital, 33 (82.5%) had positive RNA scores. Among the seven ‘missed’ admissions, five were ultimately diagnosed as anatomical trauma (sigmoid volvulus, bowel obstruction, or ovarian torsion), one was diagnosed as acute diverticulitis, and one was asked to return because of a false positive blood culture (investigated with a subsequent negative culture). Where the primary cause for admission was a potentially infectious disorder, 33 of 34 cases (97%) had positive RNA scores. The AUCs for the biomarkers as predictors of hospital admission were calculated to be 0.78; DEFA1, 0.52; IL8RB, 0.74; and, ALPL + IL8RB, 0.77 (Table 3).

Finally, the two cases that required surgery but were considered normal by RNA were ultimately diagnosed as bowel obstruction and ovarian torsion, both of which are probably purely mechanical causes of pain without any infectious etiology, thereby explaining the normal RNA scores.

## Discussion

Because there are currently no well-established biomarkers for undifferentiated abdominal pain, the premise of this study was that certain mRNA biomarkers may accurately identify infectious causes of abdominal pain and predict major outcomes of Emergency Department patients. mRNA biomarkers have shown promise in a variety of conditions including monitoring organ transplant rejection, cancer recurrence, Parkinson’s disease, coronary artery disease, development of severe COVID-19 symptoms, and discrimination between bacterial and viral infections^8–13^. All of the biomarkers tested in this study were chosen based on preliminary data that demonstrated accuracy for detecting appendicitis^7^.

While most traditional biomarkers are serum proteins, whole blood mRNA has several potential advantages. For infections of the gastrointestinal tract (acute appendicitis, acute cholecystitis, diverticulitis, and peritonitis), bacterial infiltration of the intestinal walls leads to an inflammatory response that involves immune cells such as neutrophils, granulocytes, macrophages, and mast cells as well as their mediators. mRNA that is elevated as a part of this immune response may be detected earlier than protein biomarkers, and is less prone to the complex processes of secretion, binding, and clearance of circulating inflammatory proteins. Biomarkers based on serum proteins or mRNA both offer advantages over CT scans, blood cultures and PCR tests because biomarkers involve no radiation exposure, can have turnaround times less than a day (and potentially less as the technology develops), and are unlikely to create false positive results due to contaminants.

Of the three RNA biomarkers, the composite of ALPL + IL8R appeared to have the highest accuracy to identify infectious causes and serious outcomes in undifferentiated abdominal pain patients. As a continuous variable, the AUC for ALPL + IL8R was 0.77 for hospital admission and 0.76 for surgery, values which are considered to demonstrate excellent discriminatory capabilities. When combined with DEFA1 > 10%, the positive predictive value (PPV) becomes more accurate: 0.86 and 0.83 to predict hospital admission and surgery, respectively. The strong association with antibiotic administration suggests, albeit retrospectively, that RNA testing could help predict if a patient would benefit from antimicrobial treatment before a final diagnosis is made. However, a rapid point-of-care test may be needed to affect real-time Emergency Department decision making.

A single biomarker is unlikely to function well for the multiple heterogeneous causes of undifferentiated abdominal pain. In addition to variation across conditions, there may be variation within a single condition, such as an ovarian cyst that becomes infected^14,15^. Furthermore, many diseases that cause abdominal pain are in a dynamic state. For example, appendicitis symptoms at 12 h may look different at 48 h. mRNA, on the basis of disease progression, is still unknown.

Combining individual biomarkers may lead to RNA “fingerprints” specific to individual diseases that cause abdominal pain. And, the mRNA “fingerprint” is yet to be determined. For example, using the ALPL + IL8RB pre-set threshold of 20%, we detected nine out of 10 cases of appendicitis. Yet, the one missed case was positive for DEFA1. Using the combined criteria of either ALPL/IL8RB > 20% or DEFA1 > 10%, all 100% of cases of appendicitis were detected. Further study is underway to assess whether a combined score using multiple RNA biomarkers is the most accurate method for predicting certain abdominal infections. This study helps us with the understanding of relationship between mRNA.

Undifferentiated abdominal pain is challenging for the Emergency Department physician and there is great pressure to perform a CT scan to rule out dangerous causes. A low-risk rule-out blood test could reduce the number of CT scans ordered in patients with abdominal pain. In addition, an accurate biomarker may assist with ruling out dangerous etiologies in patients who have recurrent visits for abdominal pain. It is possible that a biomarker could diagnose an acute abdomen in patients who did not have a diagnostic CT performed. Recently published guidelines by the Society of Academic Emergency Medicine (SAEM) bring attention to the challenge and potential harms in working up of recurrent low risk abdominal pain^16^.

The strengths of this study are its prospective nature, its clinical relevance by enrolling patients based on complaint and not final diagnosis (i.e., the dimensional and heterogenous nature of the sample), its adherence to STARD guidelines, and the blinding between the results of the index test and the clinical assessment. Limitations include the use of a small convenience sample, inequivalence in sex distribution, lack of a gold standard for what defines an infectious cause of abdominal pain, test thresholds that were based on limited preliminary data, and subjectivity by clinicians regarding whether a patient undergoes surgery or hospital admission. In addition, we are limited in our ability to compare fairly the RNA biomarkers to existing biomarkers such as WBC because they are currently used in clinical practice, and thus, unable to be separated from outcome measures. Other potential sources of bias such as partial verification bias, differential verification bias, or spectrum bias could artificially inflate the accuracy estimates. Future studies will address these weaknesses in a variety of practice settings. In addition, future studies will explore serial measurements for these indicators to assess how the trends correlate with clinical course. As with all diagnostic tests, we hope that better diagnostic testing will increase appropriate downstream testing as needed, but there is a risk that additional testing leads to more testing and associated problems. Ultimately, a combination of traditional biomarkers plus novel biomarkers such as mRNA might provide the highest diagnostic yield and clinical benefit.

### Supplementary Information

Supplementary Information 1.

Supplementary Information 2.

Supplementary Information 3.

## Data Availability

The datasets used and/or analyzed during the current study available from the corresponding author on reasonable request.
