# Publisher Correction: Diagnostic accuracy of novel mRNA blood biomarkers of infection to predict outcomes in emergency department patients with undifferentiated abdominal pain

**DOI:** 10.1038/s41598-023-48752-8

**Published:** 2023-12-11

**Authors:** Andrew C. Meltzer, Richard S. Wargowsky, Seamus Moran, Tristan Jordan, Ian Toma, Tisha Jepson, Shiyu Shu, Yan Ma, Timothy A. McCaffrey

**Affiliations:** 1 Department of Emergency Medicine, School of Medicine and Health Sciences, The George Washington University Medical Center, Washington, DC 20037 USA; 2 Division of Genomic Medicine, Department of Medicine, The George Washington University Medical Center, Washington, DC 20037 USA; 3 True Bearing Diagnostics, Washington, DC 20037 USA; 4 Department of Biostatistics, The George Washington University Milken School of Public Health, Washington, DC 20037 USA; 5 Department of Biostatistics, University of Pittsburgh, Pittsburgh, PA 15260 USA

Correction to: *Scientific Reports* 10.1038/s41598-023-29385-3, published online 09 February 2023

The original version of this Article contained an error in Figure 3, where some blue bars were not rendered correctly. The original Figure 3 and the accompanying legend appear below.

**Figure 3:**
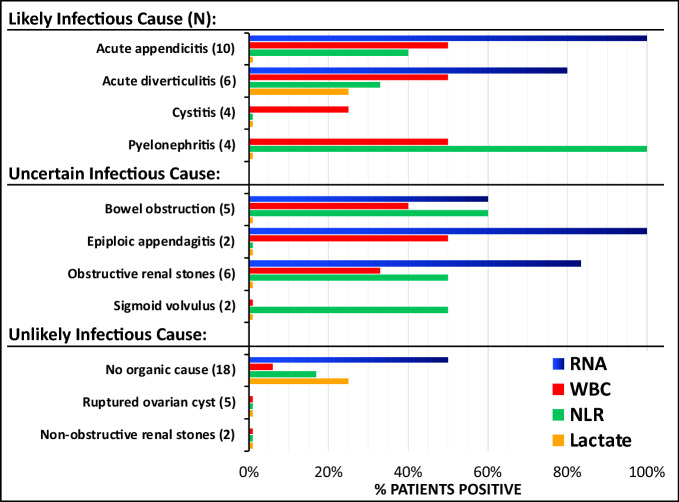
Comparison of RNA biomarkers with traditional biomarkers (WBC, NLR, Lactate). The clinical, imaging, and laboratory values of patients presenting with abdominal pain at the Emergency Department was used to categorize the patients into the diagnostic groups shown on the Y-axis. The percent of those patients that were positive for the novel RNA biomarkers DEFA1 > 5% or ALPL + IL8RB > 20% (BLUE bars), WBC count > 10 k/μL (RED bars), neutrophil/lymphocyte ratio > 6 (NLR, GREEN bars), and lactate levels > 2 mM in plasma (YELLOW bars) are shown on the horizontal axis. Lactate was not ordered on all patients, but only one diagnosis (obstructive renal stones) had no cases where lactate was ordered.

The original Article has been corrected.

